# Interpenetrating Hydrogel Networks Enhance Mechanical Stability, Rheological Properties, Release Behavior and Adhesiveness of Platelet-Rich Plasma

**DOI:** 10.3390/ijms21041399

**Published:** 2020-02-19

**Authors:** Roberta Censi, Cristina Casadidio, Siyuan Deng, Maria Rosa Gigliobianco, Maria Giovanna Sabbieti, Dimitrios Agas, Fulvio Laus, Piera Di Martino

**Affiliations:** 1 School of Pharmacy, University of Camerino, Via. S. Agostino 1, 62032 Camerino (MC), Italy; cristina.casadidio@unicam.it (C.C.); siyuan.deng@unicam.it (S.D.); maria.gigliobianco@unicam.it (M.R.G.); piera.dimartino@unicam.it (P.D.M.); 2 School of Biosciences and Veterinary Medicine, University of Camerino, Via Gentile III da Varano, 62032 Camerino (MC), Italy; giovanna.sabbieti@unicam.it (M.G.S.); dimitrios.agas@unicam.it (D.A.); 3 School of Biosciences and Veterinary Medicine, University of Camerino, Via Circonvallazione 93/95, 62024 Matelica (MC), Italy; fulvio.laus@unicam.it

**Keywords:** thermosensitive hydrogels, tissue repair, mechanical properties, rheological behavior, interpenetrating polymer networks, growth factor release

## Abstract

Platelet-rich plasma (PRP) has attracted much attention for the treatment of articular cartilage defects or wounds due to its intrinsic content of growth factors relevant for tissue repair. However, the short residence time of PRP in vivo, due to the action of lytic enzymes, its weak mechanical properties and the consequent short-term release of bioactive factors has restricted its application and efficacy. The present work aimed at designing new formulation strategies for PRP, based on the use of platelet concentrate (PC)-loaded hydrogels or interpenetrating polymer networks, directed at improving mechanical stability and sustaining the release of bioactive growth factors over a prolonged time-span. The interpenetrating hydrogels comprised two polymer networks interlaced on a molecular scale: (*a*) a first covalent network of thermosensitive and biodegradable vinyl sulfone bearing p(hydroxypropyl methacrylamide-lacate)-polyethylene glycol triblock copolymers, tandem cross-linked by thermal gelation and Michael addition when combined with thiolated hyaluronic acid, and (*b*) a second network composed of cross-linked fibrin. The PC-loaded hydrogels, instead, was formed only by network (*a*). All the designed and successfully synthesized formulations greatly increased the stability of PRP in vitro, leading to significant increase in degradation time and storage modulus of PRP gel. The resulting viscoelastic networks showed the ability to controllably release platelet derived growth factor and transforming growth factr β1, and to improve the tissue adhesiveness of PRP. The newly developed hydrogels show great potential for application in the field of wound healing, cartilage repair and beyond.

## 1. Introduction

**Scheme 1:**
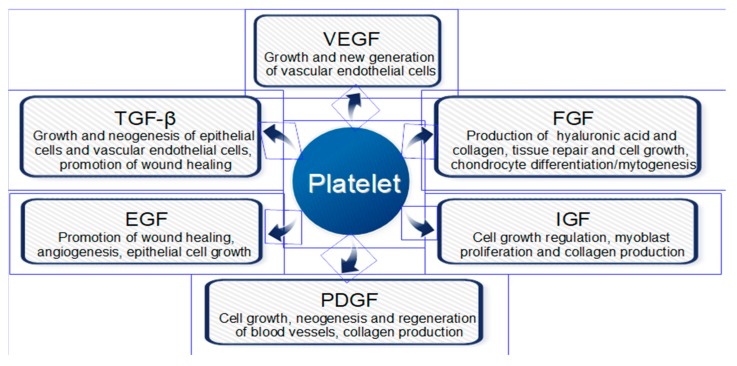
Growth factors secreted by platelets including platelet-derived growth factor, fibroblast growth factor, transforming growth factor beta, epithelial growth factor, and vascular endothelial growth factor.

In the past decade, articular cartilage injuries and their medical treatment have drawn the attention of researchers, yet remaining challenging issues to solve. The low intrinsic repair capacity of cartilage, mainly induced, among other factors, by the lack of vasculature, precludes the access of blood derived healing factors to the injure site, resulting in loss of activity, pain and disability even for small traumas. The current therapies relying on autograft and allograft implants have found some limitations. The former involves the use of tissue explanted from other body districts and re-implanted in the cartilage defects site, but the size of the explanted tissue is limited and the surgery can run into complications at both donor and acceptor sites [1]. Allograft implants require tissues from other patients. This technique also has important limitations, such as the risk of infections, disease transmission, and immune response [1]. The field of tissue engineering emerged to fill up all the drawbacks of cartilage repair and meet the urgent need for alternative healing strategies. Tissue engineering is an interdisciplinary field combining biological sciences and engineering directed to the development of tissue substitutes that restore, maintain, or enhance tissue function [2]. In tissue engineering approaches, cells and bioactive molecules are generally incorporated into biomaterial scaffolds, which can provide a suitable environment for cell-based tissue regeneration [3]. Hydrogels have emerged as particularly promising materials for tissue engineering, as they can act simultaneously as scaffolding materials for cells and/or releasing matrices for biologically active and cell modulating substances. Their water content, soft nature, and porous structure mimic biological tissues, and make them suitable to accommodate cells and to encapsulate and release water-soluble compounds like proteins in a controlled fashion [4]. In our previous work, we developed a new rapidly in situ gelling and biodegradable hydrogels based on hyaluronic acid and poly(hydroxypropyl methacrylamide lactate)-polyethylene glycol (p(HPMAm-lac)-PEG) triblock copolymers [5] and assessed its cytocompatibility in vitro with mesenchymal stem cells and fibroblasts, as well as its biocompatibility in vivo [6]. The developed hydrogels were demonstrated to possess the capability to controllably release and protect from degradation a number of model and therapeutic proteins, [7,8] as well as to encapsulate viable cells for cartilage tissue engineering applications. [9] In the present study, a novel interpenetrating hydrogel network composed of two interlaced polymeric networks independently cross-linked via covalent or ionic bonds was designed and synthesized by combining the previously developed thermosensitive hybrid hydrogel with activated platelet-rich plasma (PRP) from equine source. PRP is a sample of plasma with a twofold or more increase in platelet concentration above baseline levels, that acts as a reservoir of an autologous assortment of growth factors collected in α-granules, such as platelet-derived growth factor (PDGF), transforming growth factor (TGF-β), bone morphogenetic protein (BMP), epithelial growth factor (EPG), fibroblast growth factor (FGF), and insulin growth factor I (IGF-I) (Scheme 1). Moreover, PRP contains plasma elements and proteins with several biological effects. Growth factors can stimulate cellular division, growth and differentiation, therefore can be used as modulating substances for enhancing the tissue regeneration. The application of PRP in cartilage repair is rather recent but promising. The effects of PRP in cartilage repair suggested that PRP increased chondrocyte and mesenchymal stem cell proliferation, proteoglycan deposition, and type II collagen deposition. [10] The limitations associated with the administration of PRP in vivo include poor residence time at the tissue defect site and limited exposure of cells to released growth factors. In order to prolong the release time of growth factors released by platelets and enhance the stability of PRP at the tissue defect site, strategies for the encapsulation of PRP into hydrogels have been proposed. PRP was encapsulated into dextran [11], chitosan [12], and agarose [13] -based hydrogels. Gelatin hydrogels appear the most studied systems for this application. [14,15,16] However, to the best of the authors’ knowledge, a PRP-based interpenetrating hydrogel network is proposed for the first time in this work, to the scope of enhancing PRP stability and prolonging growth factor release.

In the present work, the suitability of vinyl sulfone bearing p(HPMAm-lac)-PEG-based hydrogels for the stabilization of PRP and the controlled release of growth factors was investigated. Hydrogel formation, mechanical properties, degradation behavior and tissue adhesiveness of interpenetrating hydrogel networks were studied. Furthermore, the capability of the newly developed hydrogels to release platelet-derived growth factors in a sustained and controlled fashion was investigated. Two growth factors relevant to cartilage repair, platelet derived growth factor (PDGF-BB) and transforming growth factor β1 (TGF-β1), were selected. It is believed that both promote the healing of soft and bone tissues through stimulation of collagen production and the initiation of callus formation [17,18].

## 2. Results

### 2.1. Design and Synthesis of Polymers for the Preparation of Rapidly In-Situ Gelling Hydrogels

A vinyl sulfone modified triblock copolymer based on p(HPMAm-lac)-PEG t(VinylSulfTC_0) was synthesized by a one-pot synthesis route. In detail, 3-mercaptopropionic acid-divinyl sulfone (3-MPA-DVS) was synthesized using a large excess of DVS over 3-MPA and, subsequently, a *N,N’*-dicyclohexylcarbodiimide (DCC) coupling reaction between 3-MPA-DVS and VinylSulfTC_0 occurred. The synthesized vinyl functionalized triblock copolymer VinylSulfTC_10 displayed 10% of its free hydroxyl groups on lactate side chains modified with cross-linkable moieties. The partial modification of the triblock copolymer with vinyl sulfone moieties led to a decrease in the cloud point (CP), which dropped from 33 to 29, for VinylSulfTC_10 due to an increase in the polymer hydrophobicity. The dependence of CP on hydrophobicity was observed earlier [19], however, differently from previous results, the extent of temperature decrease is less pronounced with vinyl sulfone derivative as compared to their methacrylate and acrylate analogues. [20] Gel Permeation Chromatography (GPC) analyses revealed that MWs and polydispersity indexes (PDIs) were constant upon partial modification of the free hydroxyl groups with vinyl sulfone moieties, indicating that no premature polymerization of vinyl sulfone residues had occurred during DCC coupling reaction, workup and lyophilization procedures. Table 1 overviews the main characteristics of the synthesized polymers.

Thiolated hyaluronic acid of a molecular weight of 37.9 kDa was used as a cross-linker and synthetized with a yield of approximately 80% and a degree of substitution (DS) value of 56%. As shown in Table 2, there was good agreement between DS values calculated according to 1H-NMR and determined by Ellman’s method, indicating no premature formation of inter- and intra-chain disulfide bonds.

### 2.2. Design and Formulation of the Interpenetrating Polymer Networks

**Scheme 2:**
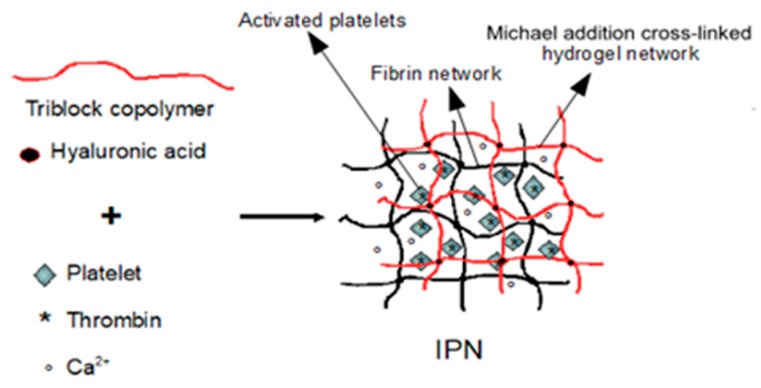
Schematic representation of interpenetrating polymer networks (IPNs) prepared by combining a vinyl sulfonated thermosensitive triblock copolymer, thiolated hyaluronan, platelet concentrate (PC), thrombin and Ca^2+^. The resulting IPN comprising a Michael addition cross-linked thermosensitive network (in red color) and a fibrin network (in black color) encapsulating activated platelets releasing growth factors.

**Figure 1:**
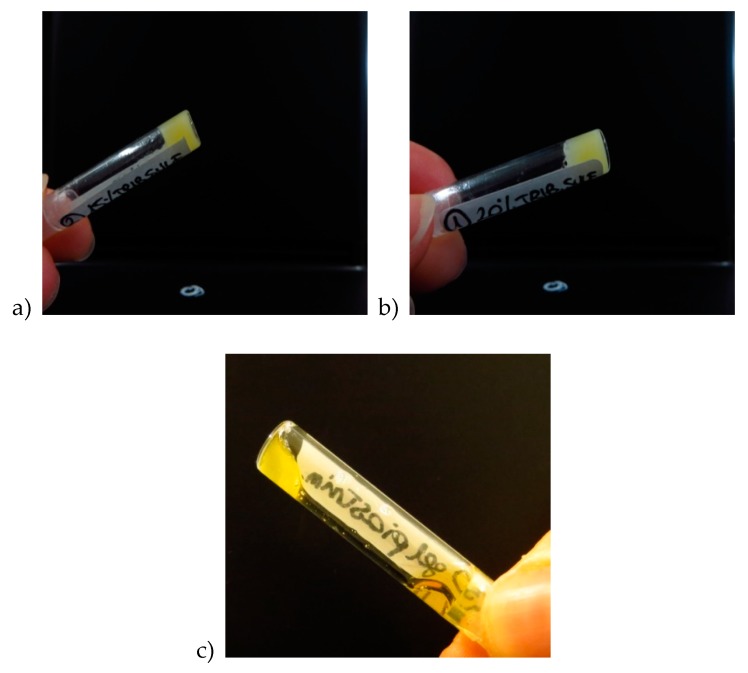
(**a**) Intepenetrating polymer networks after 10 min incubation at 37 °C for a triblock copolymer concentration of 20% w/w (20% TIPN), (**b**) intepenetrating polymer networks after 10 min incubation at 37 °C for a triblock copolymer concentration of 15% w/w (15% TIPN), and (**c**) PRP gel.

The hydrogel delivery system designed in this study for the encapsulation and controlled release of autologous growth factors is based on an interpenetrating polymer network comprising a covalently cross-linked thermosensitive and biodegradable polymeric hydrogel, interlaced on a polymer scale to a fibrin network. The resulting complex hydrogel system, schematically depicted in Scheme 2, stabilizes and encapsulates platelets and growth factors, releasing them in a sustained fashion to the target tissue over a prolonged period of time, ultimately degrading by enzymatic and hydrolytic mechanisms. For the formulation of the interpenetrating polymer networks (IPNs), vinyl sulfonated triblock copolymers VinylSulTC_10 were dissolved at different concentrations (15% and 20% w/w) in liquid PC derived from equine blood. Separately, thiolated hyaluronic acid (HA-SH) was dissolved in the clotting activators solution containing thrombin and calcium gluconate. Both polymers were stored at 4 °C until complete dissolution prior to use. The solutions were then mixed and incubated at 37 °C. Immediately after the mixing of both polymer solutions, prior to incubation to 37 °C, the system appeared as a slightly viscous material showing flow behavior. This behavior potentially allows in vivo for minimally invasive administration of the hydrogel formulation via simple injection. As expected, immediate increase in the solution viscosity was observed upon increase of the temperature up to 37 °C due to thermal gelation of the triblock copolymers. In line with expectations and previous results, higher viscosity and physical stability were observed for hydrogels of higher polymer content. The presence of a thermosensitive polymer in the newly designed hydrogel is an extremely interesting feature of the system, as it allows to potentially create a depot system able to stably entrap growth factors released from platelets, preventing undesired burst protein release and premature dissolution of the polymer chains in the physiological environment. After approximately 10 min upon incubation at 37 °C, strong viscoelastic networks stable at the bottom of the vials and displaying solid-like behavior were formed by simultaneous Michael addition cross-linking between thiol and vinyl sulfone groups and by the formation of a fibrin network triggered by the activation of PC (Figure 1a,b). This simultaneous but independent cross-linking mechanism led to the formation of an interpenetrating polymer network, where the first network is formed by Michael addition crosslinked vinyl sulfonated triblock copolymer and hyaluronic acid and the second network is formed by fibrin. Platelets supported the formation of a dense, stable fibrin network via interactions between the alphaIIbbeta3 integrin and the fibrin network, whereas tissue factor-bearing cells regulate fibrin structure and stability predominantly via procoagulant activity. Fibrin is converted from fibrinogen by the proteolytic activity of thrombin, cleaving off two different peptides from the fibrinogen backbone. The peptides are named fibrinopeptide A and B (FPA and FPB) and are present in two copies on each fibrinogen molecule. FPA is initially cleaved off at a considerably faster rate than FPB, and it has been determined that the polymerization reaction is only dependent on the FPA cleavage. The fibrin molecules assemble to form protofibrils, which are then stabilized by the secondary cleavage of FPB [21]. The described mechanism led to the formation of gellified PRP after approximately 20 min incubation at 37 °C. Figure 1c shows that platelet encapsulating fibrin gel (derived for the thrombin activation of PRP) possessed a lower mechanical stability as compared to PRP-based interpenetrating hydrogel networks and the resulting fibrin network showed a remarkable extent of shrinking with expulsion of part of its aqueous content, which was not observed when formulated into interpenetrating hydrogel networks.

### 2.3. Rheology

**Figure 2:**
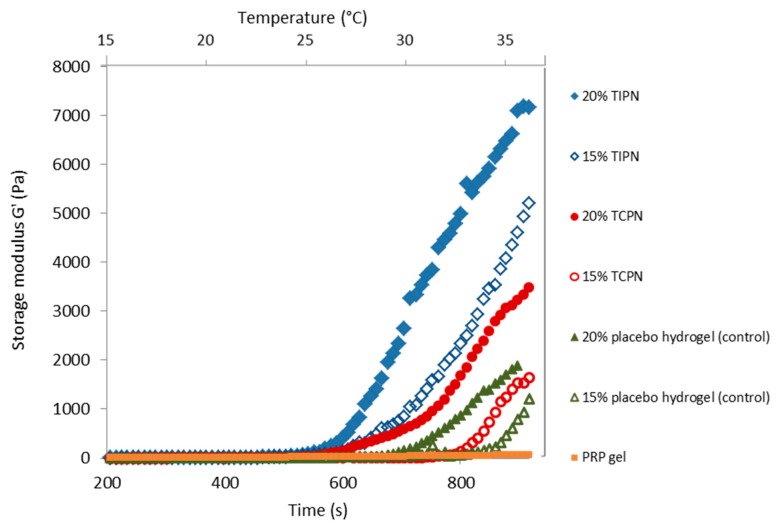
Comparison of storage (G’) moduli as a function of time for different hydrogel formulations.

**Figure 3:**
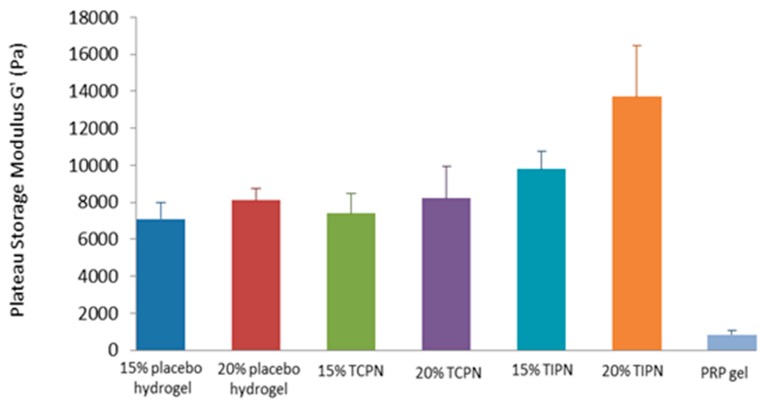
Comparison of plateau storage (G’) moduli for different hydrogel formulations.

**Figure 4:**
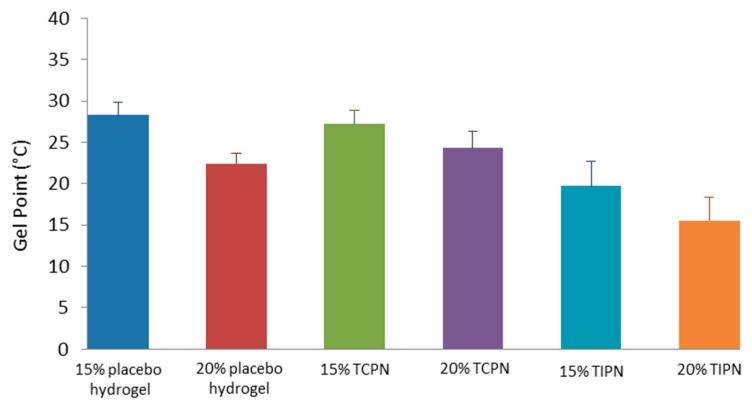
Comparison of gel point temperatures (°C) for different hydrogel formulations.

Rheological characterization was performed by temperature sweep analysis from 18 °C to 37 °C followed by time sweep analysis at the constant temperature of 37 °C to the aim of investigating the variation of gel mechanical strength in time during gelation. Figure 2 shows the comparison of storage modulus (G’) for hydrogel formulations, differing in polymer composition, triblock copolymer concentration and type of crosslinking method. Among the tested formulations, PRP gels displayed, as expected, the weakest mechanical properties, reaching very low values of storage modulus. Placebo Michael addition crosslinked thermosensitive hydrogels showed higher G’ compared to PRP gels, with the hydrogel containing 20% w/w triblock copolymer content showing a faster and higher increase in G’ compared to 15% w/w triblock copolymer hydrogels. These data lead to the conclusion that hybrid hydrogels crosslinked by covalent bonds (Michael addition) possess superior rheological and mechanical properties compared to natural physical hydrogels. When thermosensitive Michael addition hydrogels are loaded with platelet concentrate (PC) (20% TCPN and 15% TCPN), a further increase in G’ was observed, indicating that the high titer of platelets loaded into the hydrogel contributed to enhance the stiffness of the formulation. When the loaded PC is activated by thrombin and Ca^2+^ ions, interpenetring networks of different triblock copolymer concentrations are formed (20% TIPN and 15% TIPN). The coexistence of two interlaced polymer networks in the same hydrogel system gave the most significant contribution to the enhancement of the rheological properties of PRP, indicating a synergistic effect on improving mechanical behavior when the two polymer networks are interpenetrating. If we compare the plateau storage modulus values for all hydrogel networks (Figure 3), we can easily conclude that the formulation of PRP into injectable thermosensitive and Michael addition crosslinked hydrogels is an effective method to dramatically enhance the mechanical properties of PRP gel, with an increase in plateau G’ up to 7 folds compared to PRP gel value. Figure 4 shows the gel point temperatures of the tested formulations, defined as the temperature at which G’ equals G’’ (see Figure S1 in Supplementary Section). In line with rheological behavior, gel point data clearly highlight that viscoelastic networks were formed at lower temperatures when the hydrogels contained a higher polymer content and when they were composed by interpenetrating networks. In Figure 4, the gel point temperature for PRP gels is not reported because the loss modulus (G’’) always dominated the storage modulus (G’) in the considered time-span for the analysis. This implies that PRP gels behave as liquid-like fluids.

**Figure 5:**
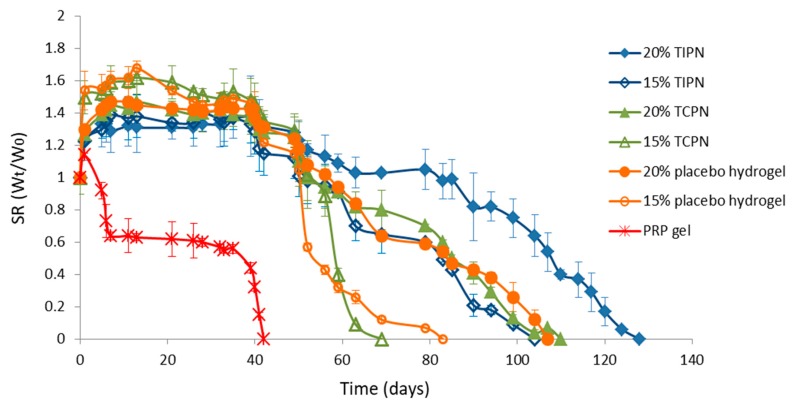
Comparison of degradation behavior (expressed as swelling ratio SR) between different hydrogel formulations.

The hydrogels were degraded under physiological conditions. When the hydrogels were formed, PBS buffer was added on top of their upper surface, and the hydrogels were incubated in the presence of PBS buffer at pH 7.4 at 37 °C. Figure 5 shows the comparison between the degradation profiles of different hydrogel formulations expressed as swelling ratio (SR) as a function of time. It can be observed that PRP gels displayed the shortest degradation time, completely dissolving in medium after 60 days and showing negligible swelling behavior. Conversely, both placebo and TCPN hydrogels showed swelling behavior, with maximum SR values of 1.5 and 1.8 for 15 and 20% w/w hydrogels, respectively. This behavior indicates that the placebo and TCPN hydrogels possess a more flexible structure and a higher capacity of water uptake as compared to fibrin networks. Interestingly, both placebo and TCPN hydrogels showed very similar degradation profiles, indicating that the presence of PC within the network, although contributing to increase the storage modulus of the system, did not have significant effect in the degradation behavior. TIPN networks showed the highest stability in physiological buffer, with a degradation time varying from 105 to 130 days, for 15% w/w and 20% w/w polymer content, respectively. Compared to TCPN hydrogels, lower SR values were achieved, possibly because the interpenetrating structure made the network more rigid, immobilizing the polymeric chains and making them less capable to absorb water. The higher stability of TIPNs compared to TCPNs can be ascribed to the higher crosslink density of the former. It is possible to envision that when applied in vivo, the degradation of these hydrogels will be accelerated by the presence of fibrinolytic enzymes and for the presence of hyaluronidases. Taken together, these data showed that the formulation of PRP into thermosensitive hydrogels greatly enhanced the stability of PRP, making it potentially suitable for cartilage tissue engineering application, where long-lasting residence time and bear-loading capacity are highly required.

Two growth factors relevant to cartilage repair, platelet derived growth factor (PDGF-BB) and transforming growth factor β1 (TGF-β1), were selected. It is believed that both promote the healing of soft and bone tissues through stimulation of collagen production and the initiation of callus formation [17].

**Figure 6:**
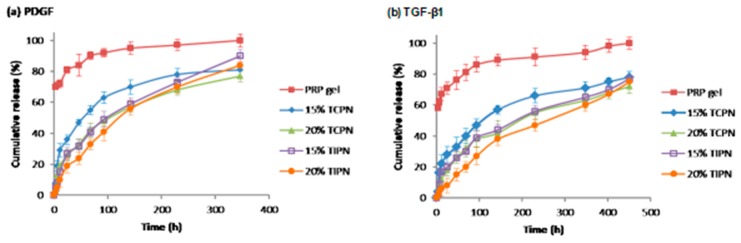
Release kinetics for (**a**) PDGF and (**b**) TGF-β1 from different hydrogel formulations.

The common preparation method for PRP gels is through thrombin activation. However, the thrombin can totally break up platelets, which leads to the burst release of encapsulated growth factors and decreases the therapeutic efficacy of PRP on a prolonged time-span. To investigate whether the formulation of PRP into thermosensitive hydrogels can circumvent the rapid and uncontrolled release of growth factors, ELISA was used to test the release kinetics of the previously mentioned growth factors. As shown in Figure 6, for both proteins tested PRP gels showed a significant burst release ranging from 60% of TGF-β1 to 70% of PDGF. Hydrogel formulations dramatically reduced the burst release, releasing PDGF with a release mechanism that is mainly diffusional, while TGF-β1 followed first order release kinetics for 15% TCPN and almost linear release for 20% TIPN. The differences observed in release kinetics between the two proteins can be ascribed to their different molecular weight.

### 2.4. Tissue Adhesive Strength

**Figure 7:**
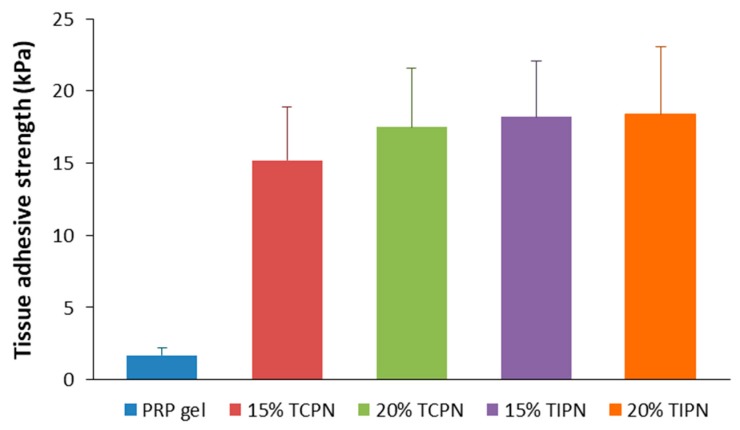
Tissue adhesive strength of PRP gel compared to hydrogel formulations of PRP at different triblock copolymer concentrations.

The clinical application of PRP generally requires its preparation in vitro and subsequent administration on target site. It is normally challenging to tightly fix PRP, because of its low tissue adhesiveness. Lap-shear measurements confirmed the low adhesive strength of PRP (Figure 7) and revealed that when PRP is combined into both TCPNs and TIPNs the tissue adhesive strength significantly increased, reaching values from 15 to approximately 18 kPa. The improvement in tissue adhesiveness may be due to the crosslinking mechanism of the thermosensitive hydrogel, that is based on the addition of nucleophile groups to α, β unsaturated groups (vinyl sulfones). It is likely that at the interface with tissues, the nucleophile acceptors may cross-react with nucleophiles present on biological tissues (for example amine residues of proteins), leading to engraftment of the hydrogel to the adjacent tissue.

## 3. Discussion

PRP is a widely used autologous source of growth factors, clinically relevant for the repair of a variety of tissues, including cartilage, skin, tendons and bone. Compared to the use of recombinant growth factors, PRP is from autologous source, therefore it can be used without the risk of immunological reactions and it is a cost-effective therapy. It has been demonstrated that the unique cocktail of growth factors and anti-inflammatory cytokines of PRP induces cell recruitment and proliferation, ultimately leading to tissue repair. [22,23] In common clinical practice, PRP is administered by means of local injection. However, the retention of PRP at the target site is very poor and the need for repeated administration reduces efficacy and patient compliance. Alternatively, PRP can also be used in the gel state, upon activation by thrombin and Ca^2+^ ions. However, the resulting hydrogel, composed of scaffolding fibrin, has poor mechanical properties, that are instead of paramount importance, especially when PRP is applied in mechanically demanding environments, such as the joints or bones. Additional drawbacks associated to use of PRP gel are: (i) the burst release of growth factors and cytokines during thrombin activation, that may cause tissue hyperplasia [24], (ii) rapid enzymatic degradation in vivo by proteases, especially when PRP gel is loaded with cells [25], and (iii) weak tissue adhesiveness, leading to poor bio-integration. All these drawbacks contribute to the reduction of the time of action and efficacy of PRP. The challenge we aimed to face in this work is to develop a strategy to sustain the beneficial biological effect of PRP at a desired site of action over a prolonged period of time, using a minimally invasive administration method. A number of authors successfully encapsulated PRP into biodegradable but mechanically stable hydrogel materials. For example, chitosan scaffolds were proposed for PRP encapsulation and growth factor controlled release [12]. Similarly, PRP loaded scaffolds of hyaluronic acid/gelatin or gelatin were investigated for bone healing [3,26]; the same clinical application was proposed for PRP loaded collagen scaffolds [27], while gelatin hydrogels were developed for meniscal regeneration [28]. Although these approaches alleviate growth factors burst release and unfavorable mechanical properties of PRP, they are based on the use of preformed scaffolds, which imply surgical administration. Injectable counterparts were studied by Pei et al., who presented a tricalcium phosphate/chitosan hydrogel for PRP loading [29] and by Tabata et al., who developed gelatin microhydrogels [30]. These technologies use crosslinking agents that may potentially leach out of the gel upon administration if gelation kinetics is slow or clog the needle during administration if kinetics is too fast or, in case of microhydrogels, they are difficult to be prepared in the clinics for the direct encapsulation of a patient’s PRP. In situ photo-crosslinkable materials [31] and enzyme-crosslinked hydrogels [11] have been also considered, however the need to take light source deep in the body, the development of free radicals during crosslinking and the use of peroxidase pose some limits in the clinical application of such technologies. More recent efforts in the modification of PRP by hydrogels were made by Santana et al., who developed semi-IPNs by mixing autologous Leucocyte derived-PRP and non-crosslinked HA under controlled conditions, obtaining systems with tunable mechanical properties according to HA molecular mass [32]. In a different approach, Sell et al., designed injectable alginate hydrogels, where PRP is chemically bound to the saccharide backbone by carbodiimide chemistry [33]. Physical thermosensitive hydrogels based on PEG-poly(ethylene glycol) polymers were used in vivo in combination with PRP for the treatment of anterior cruciate ligament partial tear. The study showed increased expression of vascular endothelial growth factor and pro-collagen I was detected after PRP-hydrogel treatment [34].

In this work we propose for the first time the encapsulation of PRP into an injectable, chemically crosslinked, thermosensitive and biodegradable hydrogel that recapitulates a number of appealing properties, making this technology superior to the ones presented so far and reviewed in this section:

The hydrogel relies on a dual cross-linking method: thermosensitivity, that assures injectability of the system and immediate physical gel formation at the injection site preventing leachates and Michael addition, that chemically stabilizes the hydrogel structure, assuring prolonged residence time of the gel and sustained release of the gel payload [5].

Michael addition crosslinking is a biocompatible reaction that occurs under physiological conditions without the need for toxic catalysts and the crosslinker is a chemically modified natural polymer (thiolated hyaluronic acid) that has slow diffusive coefficient within the thermosensitive network, therefore does not disperse from the injection site.

The two polymers composing the hydrogel can be stored in the dry form and extemporaneous formulations of PRP loaded hydrogels can be easily prepared in clinical settings.

The proposed hydrogel already showed promising results in the field of protein release and tissue regeneration [6,7,8,9].

In this work we extended the application of this hydrogel technology, demonstrating its feasibility of PRP encapsulation via two different methods, that led to the formation of a PRP loaded thermosensitive and chemically crosslinked polymer network (TCPN) and a thermosensitive interpenetrating polymer network (TIPN). The latter demonstrated the highest stability in vitro and highest stiffness. The co-dissolution of a complex matrix like PRP with hydrogel precursors did not alter the formation of the hydrogels, for both preparation methods used. Moreover, compared to PRP gel, both hydrogel preparations demonstrated enhanced mechanical properties and prolonged degradation time compared to PRP gel, with the interpenetrating network showing the highest stability. Overall, the simple encapsulation of PRP into hydrogels resulted in a hydrogel system with physical, mechanical, and degradation behavior similar to the placebo hydrogel. Conversely, when PRP is not only loaded onto the hydrogels, but also activated by Ca^2+^ and thrombin, the formation of fibrin scaffold favorably influenced the mechanical stability of the hydrogel. The synergistic effect of the interlaced fibrin/triblock copolymer/HA networks, allowed the formation of hydrogels displaying storage modulus values significantly higher compared to PRP gels and placebo hydrogels. This characteristic is particularly important in cartilage/bone tissue regeneration, where load-bearing capacity is crucial for the efficacy. Additionally, the presence of the fibrin network led to a lower swelling extent compared to placebo hydrogels and longer degradation kinetics. The obtained results also indicated that storage moduli, degradation kinetics and gel point temperatures can be easily tuned by the triblock copolymer content, making the designed hydrogel system particularly adaptable to a wide range of medical needs.

Importantly, the studies on PDGF-BB and TGF-β1 release from PRP laded hydrogels revealed that the burst release of growth factors observed in PRP gels was totally inhibited by the encapsulation into the hydrogels. Both PRP loaded TCPN and TIPN showed continuous and sustained release of the selected biological cues. The release mechanism is mostly diffusional and the interpenetrating network showed the slowest release kinetics most likely due to the higher crosslink density.

Interestingly, the TCPN hydrogel loaded with non-activated PRP showed a growth factor release behavior similar to TIPN hydrogels, where the presence of thrombin and Ca^2+^ triggered the pre-activation of platelets. This observation indicates that the hydrogel precursors, most likely hyaluronic acid, were responsible for the activation of platelets and subsequent release of growth factors. This hypothesis is confirmed in a study by Ishibashi et al., who demonstrated the activation of platelets with release of growth factors when PRP is combined with HA. [35] Similarly, an earlier report by Chen et al. showed a synergistic effect of HA and PRP on cartilage tissue repair. [36]

## 4. Materials and Methods

### 4.1. Materials

Unless indicated otherwise, all the chemicals were obtained from Sigma-Aldrich (Stenheim, Germany) and were used as received. Sodium hyaluronate with a molecular weight of 37900 Da was supplied by LIFECORE BIOMEDICAL, LLC (Chaska, Minnesota, USA). ELISA kits were purchased from Bioptis (Liege, Belgium). HPMAm-monolactate and HPMAm-dilactate were synthesized according to a previously reported method [37]. The synthesis of triblock copolymers with PEG as middle block and polyHPMAmlactate as outer blocks was described previously [38]. 3,3′-dithiobis(propanoic dihydrazide) (DTP) was synthesized by the method described by Vercruysse et al. [39].

### 4.2. Synthesis of (Vinyl Sulfonate) Triblock Copolymer

A triblock copolymer composed of a central PEG chain of a molecular weight of 10 kDa flanked at both sides by thermosensitive side chains of HPMAm-lac_1_ and HPMAm-lac_2_ copolymerized at a 1:1 molar ratio was synthesized by free radical polymerization as described by Vermonden et al. [38]. This triblock copolymer, indicated as VinylSulTC_0 and displaying thermosensitive behavior in aqueous solutions, was subsequently derivatized with vinyl sulfone moieties, according to the synthetic route reported in Chapter 4 of the resent thesis, in order to introduce chemically cross-linkable sites. The degree of substitution (DS) of vinyl sulfone groups was defined as percentage of the free OH-groups that have been modified. Vinyl sulfone bearing polymer are indicated as VinylSulTC_n, where n is the DS. The vinyl sulfone modified triblock copolymer was characterized by ^1^H-NMR, GPC and light scattering. The DS was determined by ^1^H-NMR, as previously described. [8] 

Before Vinyl sulfonation. ^1^H-NMR, DMSO-d_6_, δ in ppm: 7.3 (1H, -N*H*CH_2_CHCH_3_), 5.5-5.2 (1H, -O*H*CHCH_3_), 5.0-4.8 (2H, -NHCH_2_C*H*(CH_3_)O and -COC*H*(CH_3_)O), 4.2-4.1 (1H, -COC*H*(CH_3_)OH ), 3.5 (909 H, -OC*H_2_*C*H_2_* PEG protons), 3.1 (2H, -NHC*H_2_*), 1.5-0.8 (main chain protons).

After Vinyl sulfonation. ^1^H-NMR, DMSO-d_6_, δ in ppm: 7.3 (1H, -N*H*CH_2_CHCH_3_), 6.9 (1H, -SO_2_C*H*=CH_2_), 6.3-6.2 (2H, -SO_2_CH=C*H_2_*), 5.4-5.2 (1H, -O*H*CHCH_3_), 4.9-4.8 (2H, -NHCH_2_C*H*(CH_3_)O and -COC*H*(CH_3_)O), 4.2-4.1 (1H, -COC*H*(CH_3_)OH), 3.5 (909 H, -OC*H_2_*C*H_2_* PEG protons), 2.7 (8H, -C*H_2_*C*H_2_*SC*H_2_*C*H_2_*) 1.7-0.7 (main chain protons).

### 4.3. Synthesis of Thiolated Hyaluronic Acid (HA-SH)

Hyaluronic acid was derivatized with thiol groups, to a varying extent, slightly modifying the procedure described by Shu et al. [40]. The extent of thiol derivatization, also called degree of substitution (DS), is defined as the number of 3-3′-dithiobis propanoic hydrazide (DTP) residues per 100 disaccharide units. The typical reaction procedure for thiolate hyaluronic acid is described in Chapter The final product was obtained as a white powder after lyophilization. The DS was determined by ^1^H-NMR [5] and Ellman’s method [41]. Thiolated hyaluronic acid of different DS is indicated as HA-SH_n’, where n’ indicates the DS.

^1^H-NMR, D_2_O δ in ppm: 4.6-3.2 protons of hyaluronic acid, 2.7 (C*H_2_*SH), 2.5 (C*H_2_*CH_2_SH), 1.8 (NHCOC*H_3_*).

### 4.4. H-NMR Spectroscopy

NMR spectra were recorded with a Varian Mercury Plus 400 NMR spectrometer. The polymers were dissolved in DMSO-d_6_ or D_2_O.

### 4.5. Gel Permeation Chromatography

The weight average molecular weight (Mw), the number average molecular weight (Mn) and the polydispersity index (PDI) were determined by gel permeation chromatography (GPC) using a TSKgel G4000HHR column (TOSOH BIOSCIENCE), 7.8 mm ID x 30.0 cm L, pore size 5 μm. PEGs of defined molecular weights ranging from 106 to 1015000 Da were used as calibration standards. The eluent was THF, the elution rate was 1.0 mL/min and the column temperature was 35 °C. The samples were dissolved in THF at a concentration of 5 mg/mL.

### 4.6. Determination of the Cloud Point

The cloud point (CP) of the polymers was determined by means of light scattering, using a Zetasizer Nano-S90, Malvern Instruments. The samples were dissolved at a concentration of 3–5 mg/mL in ammonium acetate buffer 120 mM pH 5.0 in order to minimize the polymer hydrolysis. Light scattering measurements were performed at a fixed scattering angle of 90° during temperature ramps from 5 to 40 °C, at a heating rate of 1 °C/min. The CP was determined as the onset of increasing light scattering intensity.

### 4.7. Preparation of PC

Harvesting of whole blood from the horse vein directly was accomplished using a syringe containing 3.8% citrate solution sterilely from a pharmaceutical preparation. Under a laminar flow hood, the blood from the syringe is transferred gently to a 50 mL centrifuge tube. Centrifugation was accomplished with a Megafuge centrifuge at 280 g for 15 min at room temperature. After the first centrifugation, the PC was carefully removed with a pipette inserted above the buffy coat and transferred to a new, sterile centrifuge tube. A second centrifugation process was then made under the same conditions. Following the second centrifugation, the concentrated PC was taken with a sterile syringe for further studies. When anti-coagulated blood is centrifuged, three layers appears. This is due to differences in the density of the blood components: the deep layer consists of red blood cells, the middle layer contains platelets and leukocytes, and the top layer is made up of platelet-poor plasma. The bottom part of the middle layer is also called buffy coat, which is rich in leukocytes.

### 4.8. Formulation of PRP Loaded Hydrogels

Hydrogels of a volume of 200 μL were prepared in cylindrically shaped glass vials (diameter of 5 mm) as follows. For the formulation of the hydrogels, the triblock copolymer was cross-linked by Michael addition with thiolated hyaluronic acid at 37 °C. Two concentrations of the triblock copolymers of 15 and 20% w/w were used, while of the hyaluronic acid were 3.0 and 4.0% w/w, respectively. Vinyl sulfonated/thiol groups were in a molar ratio of 1/VinylSulTC_10 was gently mixed at 4 °C with 75% w/w of PC. Separately, HA-SH_56 was dissolved in 17% w/w of thrombin and 8% w/w of calcium gluconate at 4 °C. When both polymers were completely dissolved HA-SH_56 solution was added to VinylSulf_10 solution and mixed thoroughly using a needle. Thrombin and calcium gluconate acted as activators of PC, that turned into PRP, forming a fibrin network physically encapsulating growth factor-releasing platelet. Upon incubation at 37 °C, a mechanically stable tree-dimensional network was formed due to the formation of Michael addition cross-links.

### 4.9. Swelling Test

Hydrogels loaded with PRP were covered with 900 μL of saline phosphate buffered (PBS pH 7.4, 150 mM, containing 0.02% w/vol NaN_3_ to prevent bacterial contamination) and allowed to swell. The swollen hydrogels were weighted at regular time intervals after removing the buffer. Subsequently, the buffer was refreshed. The swelling ratio (SR) of the hydrogels was calculated by the ratio between the initial hydrogel weight (W_0_) and the weight of the swollen hydrogel after exposure to buffer (W_t_), as previously described. [5]

Experiments were performed in triplicate.

### 4.10. Rheology

Rheological characterization was performed on a Physica – MCR 101 (Anton Paar) rheometer equipped with a Peltier plate and a 20 mm 1° steel cone-plate geometry. Solutions of p(HPMAm-lac)-PEG_n combined with HA-SH_n’ were applied between the cone and plate geometries and analyzed immediately upon mixing. A layer of silicone oil of viscosity of 0.05 Pas was wrapped around the edge of the conical geometry to prevent water evaporation. A temperature sweep test from 18 to 37 °C at a heating rate of 1 °C/min followed by a time sweep test at 37 °C were performed. For both experiments a frequency of 1Hz and 1% strain were used. Plateau storage modulus is defined as the maximum storage modulus value reached during time sweep tests by the hydrogels at 37 °C, upon full crosslinking. Gel point temperature is defined as the temperature at which strage modulus equals loss modulus during temperature sweep tests.

### 4.11. Growth Factors Release Kinetics

Release kinetics studies were performed to determine whether there is a sustained release of growth factors from PRP loaded hydrogels. Briefly, 200 µL PRP loaded hydrogel precursors was added into a cylindrical glass vial with screw cap. The vial was stored at 37 °C to allow hydrogel formation and, subsequently, the gel was exposed to 500 µL phosphate buffer solution (PBS) at pH 7. At predetermined time-points, the PBS was collected and refreshed for two weeks. The collected PBS was stored at −80 °C before use. Two growth factors were selected and quantified in the supernatants: TGF-β and PDGF. The quantification of growth factors was performed by enzyme-linked immune sorbent assay (ELISA) according to manufacturer’s instructions (Equine growth factors ELISA sets, Bioptis (BE)). Cumulative release of both growth factors was obtained normalizing the amount of growth factors at each time-point to the total amount present in the PRP lysate solution used. All release profiles were performed in triplicate.

### 4.12. Tissue Adhesive Strength of PRP Loaded Hydrogels

The tissue adhesive strength of PRP loaded hydrogels was quantified by lap-shear measurements. [31,42] Briefly, a piece 3.5 cm × 2.5 cm hog casing was attached to a glass slide by cyanoacrylate glue. Subsequently, 200 µL hydrogel precursors was evenly smeared on it and covered with another hog casing-coated glass slide. The samples were stored at 37 °C for the time necessary for hydrogel cross-linking. One side of each sample was fixed and pull strength was added to the other side, by adding water dropwise. When the two glass slides were separated, the mass of the water was recorded and the tissue adhesive strength was calculated according to Liu et al [31].

## 5. Conclusions

In the present work, the ability of thermally and Michael addition cross-linked vinyl sulfone bearingPEG-p(HPMA-lac)/hyaluronan hydrogels to encapsulate PRP, enhancing its stability was studied. Hydrogels were loaded with activated and non-activated PRP, leading to the formation of a PC-loaded hydrogel and an interpenetrating polymer network. From rheological and degradation studies it can be concluded that the hydrogels increased the stability in vitro and the mechanical strength of PRP gel, prolonging its degradation time from 40 to 130 days in the absence of lytic enzymes and increasing its elastic modulus to at least 15 kPa. Furthermore, the ability to controllably release growth factors, and prevent their burst release was demonstrated, as well as the enhancement of tissue adhesiveness. The developed hybrid networks hold potential as matrix for cartilage tissue engineering application, owing to prolonged residence time, superior load-bearing capacity and potential reparative effects on cartilage.

## Figures and Tables

**Table 1:** Overview of the main characteristics of VinylSulfTC_n copolymers, as determined by ^1^H-NMR, Gel Permeation Chromatography and light scattering.

Name	Mn ^†^ (kDa)	Mn ^‡^ (kDa)	Mw ^‡^ (kDa)	PDI ^‡^	Cloud Point * (°C)	Feed Molar Ratio 3-MPA/OH	Obtained DS ^†^ (%)	Yield (%)
VinylSulTC_0	47	25.7	52.5	2.04	33	0	0	60
VinylSulTC_10	54	25.7	54.2	2.1	29	0.20 ^†^	10.7	98

† based on ^1^H-NMR, ‡ based on GPC using Polyethylene glycol standards, * based on light scattering.

**Table 2:** Overview of the main characteristics of thiolated hyaluronic acid (HA-SH)_56, as determined by 1H-NMR and Ellman’s method.

Name	DTP Feed Ratio (%)	Obtained DS ^‡^ (%)	Obtained DS * (%)	Conversion %	Yield (%)
HA-SH_56	75	59	53 ± 4	37	82

‡ based on ^1^H-NMR, * based on Ellman’s method.

## Supplementary Materials

The following are available online at https://www.mdpi.com/1422-0067/21/4/1399/s1.

## References

[1] Giannoudis P.V., Dinopoulos H., Tsiridis E. (2005). Bone substitutes: An update. Injury.

[2] Ortinau S., Schmich J., Block S., Liedmann A., Ludwig J., Weiss D.G., Helm C.A., Rolfs A., Frech M.J. (2010). Effect of 3D-scaffold formation on differentiation and survival in human neural progenitor cells. Biomed. Eng. Online.

[3] Kim Y.-H., Furuya H., Tabata Y. (2014). Enhancement of bone regeneration by dual release of a macrophage recruitment agent and platelet-rich plasma from gelatin hydrogels. Biomaterials.

[4] Censi R., Di Martino P., Vermonden T., Hennink W.E. (2012). Hydrogels for protein delivery in tissue engineering. J. Control. Release.

[5] Censi R., Fieten P.J., Di Martino P., Hennink W.E., Vermonden T. (2010). In Situ Forming Hydrogels by Tandem Thermal Gelling and Michael Addition Reaction between Thermosensitive Triblock Copolymers and Thiolated Hyaluronan. Macromolecules.

[6] Sabbieti M.G., Dubbini A., Laus F., Paggi E., Marchegiani A., Capitani M., Marchetti L., Dini F., Vermonden T., Di Martino P. (2017). In vivo biocompatibility of p(HPMAm-lac)-PEG hydrogels hybridized with hyaluronan. J. Tissue Eng. Regen. Med..

[7] Casadidio C., Butini M.E., Trampuz A., Di Luca M., Censi R., Di Martino P. (2018). Daptomycin-loaded biodegradable thermosensitive hydrogels enhance drug stability and foster bactericidal activity against Staphylococcus aureus. Eur. J. Pharm. Biopharm..

[8] Censi R., Casadidio C., Dubbini A., Cortese M., Scuri S., Grappasonni I., Golob S., Vojnovic D., Sabbieti M.G., Agas D. (2019). Thermosensitive hybrid hydrogels for the controlled release of bioactive vancomycin in the treatment of orthopaedic implant infections. Eur. J. Pharm. Biopharm..

[9] Agas D., Laus F., Lacava G., Marchegiani A., Deng S., Magnoni F., Silva G.G., Di Martino P., Sabbieti M.G., Censi R. (2019). Thermosensitive hybrid hyaluronan/p(HPMAm-lac)-PEG hydrogels enhance cartilage regeneration in a mouse model of osteoarthritis. J. Cell. Physiol..

[10] Fortier L.A., Barker J.U., Strauss E.J., McCarrel T., Cole B.J. (2011). The Role of Growth Factors in Cartilage Repair. Clin. Orthop. Relat. Res..

[11] Teixeira L.S.M., Leijten J., Wennink J.W., Chatterjea A.G., Feijen J., Van Blitterswijk C., Dijkstra P.J., Karperien M. (2012). The effect of platelet lysate supplementation of a dextran-based hydrogel on cartilage formation. Biomaterials.

[12] Kutlu B., Tiğlı Aydın R.S., Akman A.C., Gümüşderelioglu M., Nohutcu R.M. (2013). Platelet-rich plasma-loaded chitosan scaffolds: Preparation and growth factor release kinetics. J. Biomed. Mater. Res. Part B Appl. Biomater..

[13] Yin Z., Yang X., Jiang Y., Xing L., Xu Y., Lu Y., Ding P., Ma J., Xu J., Gui J. (2014). Platelet-rich plasma combined with agarose as a bioactive scaffold to enhance cartilage repair: An in vitro study. J. Biomater. Appl..

[14] Kurita J., Miyamoto M., Ishii Y., Aoyama J., Takagi G., Naito Z., Tabata Y., Ochi M., Shimizu K. (2011). Enhanced Vascularization by Controlled Release of Platelet-Rich Plasma Impregnated in Biodegradable Gelatin Hydrogel. Ann. Thorac. Surg..

[15] Suzuki S., Morimoto N., Ikada Y. (2013). Gelatin gel as a carrier of platelet-derived growth factors. J. Biomater. Appl..

[16] Matsui M., Tabata Y. (2012). Enhanced angiogenesis by multiple release of platelet-rich plasma contents and basic fibroblast growth factor from gelatin hydrogels. Acta Biomater..

[17] Hokugo A., Ozeki M., Kawakami O., Sugimoto K., Mushimoto K., Morita S., Tabata Y. (2005). Augmented Bone Regeneration Activity of Platelet-Rich Plasma by Biodegradable Gelatin Hydrogel. Tissue Eng..

[18] Akhundov K., Pietramaggiori G., Waselle L., Darwiche S., Guerid S., Scaletta C., Hirt-Burri N., Applegate L.A., Raffoul W. (2012). Development of a cost-effective method for platelet-rich plasma (PRP) preparation for topical wound healing. Ann. Burn. Fire Disasters.

[19] Censi R., Vermonden T., Deschout H., Braeckmans K., Di Martino P., De Smedt S., Van Nostrum C.F., Hennink W.E. (2010). Photopolymerized Thermosensitive Poly(HPMAlactate)-PEG-Based Hydrogels: Effect of Network Design on Mechanical Properties, Degradation, and Release Behavior. Biomacromolecules.

[20] Censi R., Vermonden T., Van Steenbergen M., Deschout H., Braeckmans K., De Smedt S., Van Nostrum C., Di Martino P., Hennink W.E. (2009). Photopolymerized thermosensitive hydrogels for tailorable diffusion-controlled protein delivery. J. Control. Release.

[21] Wolberg A.S. (2010). Plasma and cellular contributions to fibrin network formation, structure and stability. Haemophilia.

[22] Chevallier N., Anagnostou F., Zilber S., Bodivit G., Maurin S., Barrault A., Bierling P., Hernigou P., Layrolle P., Rouard H. (2010). Osteoblastic differentiation of human mesenchymal stem cells with platelet lysate. Biomaterials.

[23] Sánchez R.A., Sheridan P.J., Kupp L.I. (2003). Is Platelet-rich Plasma the Perfect Enhancement Factor? A Current Review. Int. J. Oral Maxillofac. Implant..

[24] Carter C.A., Jolly D.G., Worden C.E., Hendren D.G., Kane C.J.M. (2003). Platelet-rich plasma gel promotes differentiation and regeneration during equine wound healing. Exp. Mol. Pathol..

[25] Pettersson S., Wetterö J., Tengvall P., Kratz G. (2009). Human articular chondrocytes on macroporous gelatin microcarriers form structurally stable constructs with blood-derived biological glues in vitro. J. Tissue Eng. Regen. Med..

[26] Son S.-R., Sarkar S.K., Linh N.-T.B., Padalhin A.R., Kim B.R., Jung H.I., Lee B.T. (2015). Platelet-rich plasma encapsulation in hyaluronic acid/gelatin-BCP hydrogel for growth factor delivery in BCP sponge scaffold for bone regeneration. J. Biomater. Appl..

[27] Sarkar M.R., Augat P., Shefelbine S.J., Schorlemmer S., Huber-Lang M., Claes L., Kinzl L., Ignatius A. (2006). Bone formation in a long bone defect model using a platelet-rich plasma-loaded collagen scaffold. Biomaterials.

[28] Ishida K., Kuroda R., Miwa M., Tabata Y., Hokugo A., Kawamoto T., Sasaki K., Doita M., Kurosaka M. (2007). The Regenerative Effects of Platelet-Rich Plasma on Meniscal Cells In Vitro and Its In Vivo Application with Biodegradable Gelatin Hydrogel. Tissue Eng..

[29] Bi L., Cheng W., Fan H., Pei G. (2010). Reconstruction of goat tibial defects using an injectable tricalcium phosphate/chitosan in combination with autologous platelet-rich plasma. Biomaterials.

[30] Saito M., Takahashi K., Arai Y., Inoue A., Sakao K., Tonomura H., Honjo K., Nakagawa S., Inoue H., Tabata Y. (2009). Intraarticular administration of platelet-rich plasma with biodegradable gelatin hydrogel microspheres prevents osteoarthritis progression in the rabbit knee. Clin. Exp. Rheumatol..

[31] Liu X., Yang Y., Niu X., Lin Q., Zhao B., Wang Y., Zhu L. (2017). An in situ photocrosslinkable platelet rich plasma – Complexed hydrogel glue with growth factor controlled release ability to promote cartilage defect repair. Acta Biomater..

[32] De Melo B.A.G., França C.G., Dávila J.L., Batista N.A., Caliari-Oliveira C., D’ávila M.A., Luzo Â.C.M., Lana J.F.S.D., Santana M.H. (2020). Hyaluronic acid and fibrin from L-PRP form semi-IPNs with tunable properties suitable for use in regenerative medicine. Mater. Sci. Eng. C.

[33] Growney E.A., Linder H.R., Garg K., Bledsoe J.G., Sell S. (2019). Bio-conjugation of platelet-rich plasma and alginate through carbodiimide chemistry for injectable hydrogel therapies. J. Biomed. Mater. Res. Part. B Appl. Biomater..

[34] Li Y., Fu S.C., Cheuk Y.C., Ong T.-Y., Feng H., Yung S.-H. (2020). The effect of thermosensitive hydrogel platelet–rich–plasma complex in the treatment of partial tear of anterior cruciate ligament in rat model. J. Orthop. Transl..

[35] Iio K., Furukawa K.-I., Tsuda E., Yamamoto Y., Maeda S., Naraoka T., Kimura Y., Ishibashi Y. (2016). Hyaluronic acid induces the release of growth factors from platelet-rich plasma. Asia-Pacific J. Sports Med. Arthrosc. Rehabilitation Technol..

[36] Chen W., Lo W.-C., Hsu W.-C., Wei H.-J., Liu H.-Y., Lee C.-H., Chen S.-Y.T., Shieh Y.-H., Williams D.F., Deng W.-P. (2014). Synergistic anabolic actions of hyaluronic acid and platelet-rich plasma on cartilage regeneration in osteoarthritis therapy. Biomaterials.

[37] Soga O., Van Nostrum C.F., Ramzi A., Visser T., Soulimani F., Frederik P.M., Bomans P.H.H., Hennink W.E. (2004). Physicochemical Characterization of Degradable Thermosensitive Polymeric Micelles. Langmuir.

[38] Vermonden T., Besseling N.A.M., Van Steenbergen M., Hennink W.E. (2006). Rheological Studies of Thermosensitive Triblock Copolymer Hydrogels. Langmuir.

[39] Vercruysse K.P., Marecak D.M., Marecek J.F., Prestwich G.D. (1997). Synthesis and in vitro degradation of new polyvalent hydrazide cross-linked hydrogels of hyaluronic acid. Bioconjugate Chem..

[40] Shu X.Z., Liu Y., Luo Y., Roberts M.C., Prestwich G.D. (2002). Disulfide Cross-Linked Hyaluronan Hydrogels. Biomacromolecules.

[41] Ellman G.L. (1959). Tissue sulfhydryl groups. Arch. Biochem. Biophys..

[42] Brubaker C.E., Messersmith P. (2011). Enzymatically Degradable Mussel-Inspired Adhesive Hydrogel. Biomacromolecules.

