# Supplementary material for: Interpenetrating Hydrogel Networks Enhance Mechanical Stability, Rheological Properties, Release Behavior and Adhesiveness of Platelet-Rich Plasma
Source: Int J Mol Sci. 2020 Feb 19;21(4):1399. doi: 10.3390/ijms21041399 (PMC7073123; doi:10.3390/ijms21041399)
Supplement: Supplementary file 1 [file ijms-21-01399-s001.pdf]

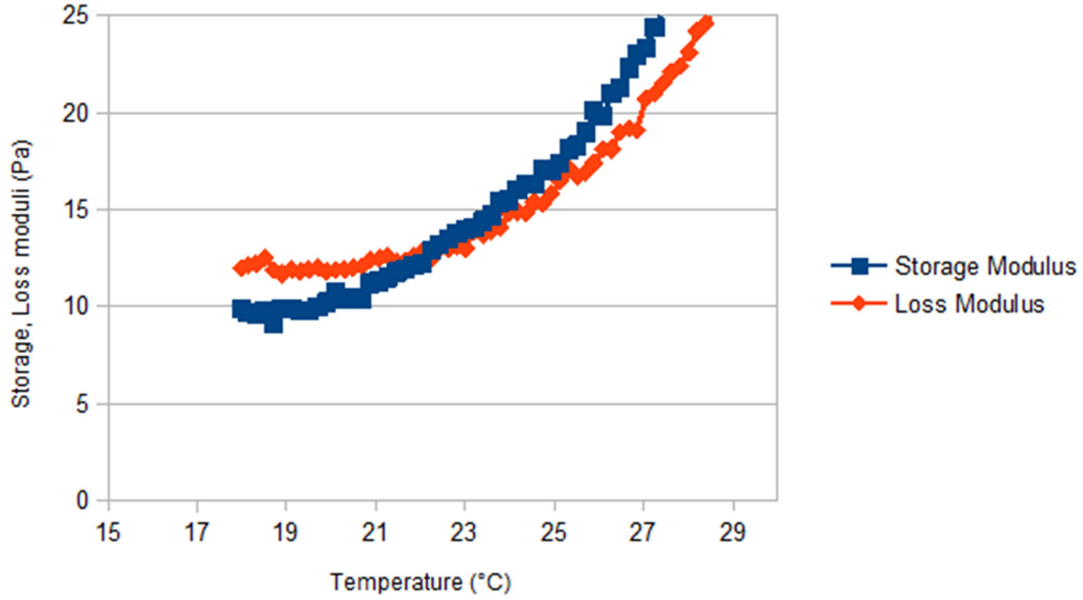

Figure S1. Representative storage and loss moduli values as a function of temperature for 20% placebo hydrogels. The figure shows that at the temperature of 22.4 °C the value of storage
